# Expression of Concern: Timing of Introduction of Complementary Foods — United States, 2016–2018

**DOI:** 10.15585/mmwr.mm7226a8

**Published:** 2023-06-30

On May 25, *MMWR* Editors were informed by authors of “Timing of Introduction of Complementary Foods — United States, 2016–2018” (*1*) that there was an overestimation in surveillance data of the proportion of children aged 1–5 years who were introduced early (age <4 months) to complementary foods. A U.S. Census Bureau April 2023 data correction technical document describes a data processing error in the National Survey of Children’s Health for data collected during 2016–2021 (*2*). Because of this, the report authors are undertaking a thorough reanalysis using the corrected data. In accordance with December 2017 guidance from the International Committee of Medical Journal Editors (*3*), this Expression of Concern notice is to alert readers that the reanalysis is currently being conducted to correct the estimates and assess the validity of conclusions in the publication. A corrected report will be published in the coming weeks.
